# Cider Vinegar

**Published:** 1858-11

**Authors:** 


### CIDER VINEGAR.

MANY persons become dyspeptic by using articles of food which are not good of their kind. Those who would avoid a most annoying disease, should make it their study not merely to have every thing properly cooked, but to have it of the very best. Vinegar is used in every family, and very much of it is prepared from unwholesome materials. Every household may protect itself from imposition by manufacturing its own vinegar, in the manner directed by the *Dubuque Northwestern Farmer*. "Soak common dried apples in water a few hours;" whether the writer means a barrel of dried apples in a pint of water as hot as melted iron, we cannot pretend to say; but, leaving the reader to find out the best way he can, in the course of several months' experiments, we proceed, remarking, in addition, that a "few hours" means two or three, or a thousand, according to circumstances. "While the apples are soaking, rub and wash them several times; remove the apples, strain the water through a tightly woven cloth; then, to a gallon of this water, add half a pint of molasses, and a piece of common brown paper," whether an inch or a mile square, we cannot say. Keep the jug, or vessel containing these ingredients, in the sun, or by a fire, and in a few days it will become cider vinegar fit for use, the apples not having been rendered unsuitable for cooking purposes by the process. While one jug is being used, another should be in preparation. The heat of the sun is preferable to that of the fire.

